# Longitudinal In Vivo 3T MRI of Naturally Occurring Early Osteochondrosis Lesions in the Piglet Humeral Epiphyseal Cartilage and Growth Plate

**DOI:** 10.1002/jor.70044

**Published:** 2025-08-10

**Authors:** Alexandra R. Armstrong, Erick O. Buko, Casey P. Johnson, Ferenc Tóth

**Affiliations:** ^1^ Department of Veterinary Clinical Sciences University of Minnesota Saint Paul Minnesota USA; ^2^ Center for Magnetic Resonance Research University of Minnesota Minneapolis Minnesota USA

**Keywords:** elbow, growth plate, osteochondritis dissecans, osteochondrosis, quantitative MRI

## Abstract

Osteochondrosis/osteochondritis dissecans (OC/OCD) is a developmental orthopedic disease primarily affecting the knee, ankle, and elbow joints of children and multiple animal species. Subclinical lesions of OC/OCD have been described, but most can be visualized only histologically in cadaveric specimens. To monitor the evolution of these lesions and to allow early separation of lesions that will undergo spontaneous healing versus requiring surgical intervention, Magnetic resonance imaging (MRI) techniques that are precise and can be used in vivo are needed. The purpose of this study was to demonstrate the utility of noninvasive 3 T MRI in the identification of naturally occurring OC lesions in the articular epiphyseal cartilage complex (AECC) and growth plate of the distal humerus in domestic piglets. *N* = 4 asymptomatic piglets underwent four consecutive, in vivo, bilateral elbow joint MRI exams under anesthesia at 4, 6, 8, and 11 weeks of age. 3D Double echo steady state (DESS) morphological images and cartilage T2 relaxation time maps were acquired using a clinical 3 T MRI scanner. After the last MRI, piglets were euthanized, and distal humeri were harvested for histologic evaluation. Multiple preclinical OC lesions were detected in the AECC and the growth plate of the examined humeri and their temporal progression or resolution was successfully monitored using MRI. Although most lesions resolved by 11 weeks of age, those remaining on MRI were confirmed histologically at necropsy.

**Clinical Significance:** In vivo 3 T MRI may allow for longitudinal monitoring of early OC lesions and determination of whether a lesion is resolving or progressing to clinical OCD that may necessitate surgical intervention.

## 1 Introduction

Osteochondrosis/osteochondritis dissecans (OC/OCD) is a developmental orthopedic disease characterized by disruption of endochondral ossification, the process of bone formation in which a cartilage template is replaced by bone during growth/development. Endochondral ossification occurs at the metaphyseal growth plates (physes), which are responsible for longitudinal bone growth, and at the articular‐epiphyseal cartilage‐complexes (AECCs), which contribute to the formation of the adult shape and size of the ends of the growing long bones (joints). OC/OCD lesions affecting the AECC have been observed in both animals and humans, whereas cases of physeal OC have only been noted in animal species [1, 2, 3, 4].

The exact etiology of OC/OCD is incompletely understood, but multiple histologic studies performed on developing joints [5, 6, 7, 8] and growth plates [1] across species have demonstrated that failure of the vascular supply to the growth cartilage is a crucial element of its pathophysiology. Indeed, unlike articular cartilage, the growth cartilage is reliant on a rich vascular network to obtain nutrients and oxygen.

Despite their shared pathogenesis, the histologic and clinical features of OC lesions in the AECC versus growth plate are different. Vascular failure to the AECC results in ischemic necrosis of the epiphyseal growth cartilage, a lesion referred to as *OC‐latens*. Conversely, ischemic injury to the growth plate is associated with the retention of viable hypertrophic cartilage [1]. Regardless, both types of lesions may progress to a focal failure of endochondral ossification [2]. In the case of the AECC, areas of necrotic epiphyseal cartilage delay the progression of the ossification front over time, resulting in a lesion that is radiographically apparent and, therefore, referred to as *OC‐manifesta*. These early OC lesions share a characteristic of having a high propensity to undergo spontaneous healing both at the AECC [9, 10, 11] and at the growth plate [1, 2, 12]. A small percentage of lesions, however, progress to clinically apparent disease. Clinical progression of *OC‐manifesta* lesions at the AECC leads to collapse of the overlying articular cartilage, resulting in the formation of a cleft extending through the articular cartilage into the underlying necrotic epiphyseal cartilage. In the absence of intervention, these lesions may develop into symptomatic osteochondral flaps or intraarticular fragments, termed *OC‐dissecans* (OCD) [3]. At the growth plate, OC lesions present with focal thickening of the growth cartilage, but typically lack the necrotic cartilage observed in AECC lesions [1]. Clinical progression of OC lesions at the growth plate in animals may result in the development of an angular limb deformity [2] or a Salter–Harris fracture [13]. Early intervention has been shown to be important in halting disease progression and improving outcomes [14, 15].

Safe and clinically relevant MRI protocols are needed to ensure that OC lesions are identified early, allowing for treatment before progression, and to facilitate assessment of healing over time. Most studies investigating the use of various MRI sequences in the early diagnosis and monitoring of OC lesions have targeted the knee joint [9, 16, 17, 18, 19], the primary predilection site of OCD in children and adolescents. The ability of novel MRI sequences to identify preclinical OC lesions in the elbow joint, the third most affected site in human patients, remains largely unexplored, although in a recent ex vivo study ultrahigh field strength (10.5 T) MRI was successfully used to identify naturally occurring *OC‐latens* and *OC‐manifesta* lesions in the distal humerus of juvenile pigs [20]. Additionally, there is a dearth of information regarding the use of MRI in the identification of OC lesions affecting the growth plate at any anatomic location.

The primary objective of the current study is to demonstrate the use of noninvasive 3 T MRI (a clinically relevant field strength) to identify naturally occurring *OC‐latens* and *OC‐manifesta* lesions in the distal humerus (elbow joint) and to investigate its ability to identify OC lesions affecting the distal humeral growth plate in domestic piglets in vivo. A piglet model was selected for this study because of the nearly 100% incidence of naturally occurring *OC‐latens* or *OC‐manifesta* lesions affecting the distal humeral predilection site in this species [21]. We hypothesized that MRI would identify preclinical OC lesions affecting the distal humeral AECC and/or the growth plate and allow monitoring of the temporal progression and/or resolution of individual lesions. To achieve this, we used a combination of morphological 3D DESS imaging and quantitative T2 mapping. While 3D DESS provides high‐resolution visualization of cartilage morphology, T2 mapping offers a sensitive measure of tissue composition, particularly water content and collagen integrity, that are altered in *OC‐latens* lesions due to chondrocyte cell death well before morphologic changes become apparent [16, 17, 18, 19, 20].

## 2 Methods

### 2.1 Animals

Four female Yorkshire cross piglets aged 4 weeks (4.4–11.8 kg) were enrolled in the study after a minimum of 3 days of acclimation with approval from the University of Minnesota Institutional Animal Care and Use Committee (#2103A‐38938). Piglets were housed in pairs for enrichment and companionship in pens that were 4 feet wide by 8 feet deep and over 6 feet tall. Water was provided ad libitum, and feed consisted of a 16% protein powdered ground corn and 20% protein ground feed twice daily, with treats (canned dog food, fig cookies, etc.) provided as needed for enrichment. The water supply was regularly tested, and representative guaranteed feed analyses were kept on file to ensure there were no known contaminants. Due to the exploratory nature of this study and the high prevalence of naturally‐occurring OC lesions in Yorkshire‐cross pigs, a sample size of four piglets was deemed sufficient to demonstrate the feasibility of using in vivo 3 T MRI to identify and track lesion progression. Future studies will include larger cohorts for quantitative analyses. It was estimated that at least 3/4 piglets would develop either *OC‐latens* or *OC‐manifesta* lesions during the study at the time points of interest, with *OC‐latens* lesions expected to develop by 6 weeks of age and *OC‐manifesta* lesions expected to be present by 10–11 weeks based on prior studies [7, 22].

### 2.2 MRI

Four piglets (*n* = 8 elbow joints) underwent in vivo imaging in a 3 T MRI scanner (MAGNETOM Prisma; Siemens Healthcare) under general anesthesia. Piglets were premedicated with a combination of Telazol (4 mg/kg), xylazine (2 mg/kg), and buprenorphine (0.02 mg/kg) administered intramuscularly and were orotracheally intubated. General anesthesia was maintained by inhalation of isoflurane vaporized in oxygen. One of the piglets (*n* = 2 elbows) was imaged at only four and 6 weeks of age to allow histological validation of the observed early lesions. The remaining three piglets (*n* = 6 elbows) were imaged at 4, 6, 8, and either 10 or 11 weeks of age. Each elbow joint was imaged individually using the same protocol, which included (i) quantitative T2 relaxation time mapping using a multi‐slice multi‐echo (MSME) spin echo sequence and (ii) high‐resolution morphological images using a 3D DESS sequence. Imaging parameters are detailed in Table 1. Quantitative T2 relaxation time maps were calculated on a pixel‐by‐pixel basis using a mono‐exponential signal decay model after removal of the first echo time image. Before generating these maps, the source T2‐weighted images were denoised using TNORDIC to improve the signal‐to‐noise ratio [23]. The regions of interest (ROIs), comprising the AECC and growth plate, were segmented using ITK‐SNAP from the 3D DESS images. The T2 maps of the ROIs were then co‐registered and overlaid on the 3D DESS images. Imaging sequences were independently evaluated by two authors (E.B., F.T.) for the presence of OC lesions. *OC‐latens* lesions were defined as discrete areas of increased T2 relaxation times within the AECC. Focal extension of the cartilage signal (bright signal consistent with adjacent cartilage on 3D DESS [24, 25, 26]) from the AECC into the subchondral bone margin was considered consistent with *OC‐manifesta*. Healing lesions were defined as cartilage signal present within the confines of the secondary ossification center. In the growth plate, OC lesions were defined as focal thickening of the growth plate that protruded into the adjacent metaphysis.

**Table 1:** MRI parameters for longitudinal imaging at 3 T.

	MSME T2 map	3D DESS
Field of view (mm)	128 × 76	128 × 76 × 48
Sampling matrix	384 × 228	384 × 228 × 160
Spatial resolution (mm^2^ or mm^3^)	0.33 × 0.33	0.33 × 0.33 × 0.30
Slices/thickness/gap (mm)	25/2.0/0.0	—
TR/TE (ms)	4500/11.5, 23.0, 34.5, 46.0, 57.5, 69.0, 80.5, and 92.0	22.9/7.5
Flip angle (degrees)	90/180	25
Bandwidth (Hz/px)	250	128
Fat sat	Yes	Yes
Acceleration	5/8 partial Fourier	6/8 partial Fourier
Scan time (min)	10:48	9:40

Abbreviation: TR/TE, repetition time/echo time.

MRI studies were reviewed by all authors together to confirm by consensus the presence of OC lesions. This consensus review took place after ARA had completed a blinded assessment of histological sections. During the review, T2 maps were used to facilitate identification and to measure *OC‐latens* lesions due to their sensitivity to compositional changes characteristic for *OC‐latens* (e.g., increased water content due to chondronecrosis). 3D DESS images were utilized to measure the overall lesion area (mm^2^) for *OC‐manifesta* and healing lesions at their greatest extent. Measurements of AECC *OC‐manifesta* lesions were limited to the retained portion of cartilage that extended into the subjacent secondary ossification center. Measurements of growth plate lesions included the full thickness of cartilage signal at areas of focal thickening of the growth plate. Digital histology images were used solely for qualitative validation.

### 2.3 Histology

Following the final MRI exam, anesthetized piglets were euthanized with intravenous phenobarbital euthanasia solution (100 mg/kg), and their distal humeri were collected for histologic assessment. Harvested specimens were fixed in 10% neutral buffered formalin for a minimum of 72 h, followed by decalcification in 10% ethylenediaminetetraacetic acid and sagittal sectioning from medial to lateral into 2 mm thick slabs (4–6 per sample). Slabs underwent standard processing and embedding, and 5‐µm thick sections were prepared for histology and stained with hematoxylin and eosin (H&E). Slides were evaluated by an experienced musculoskeletal veterinary pathologist (ARA) who was blinded to the results of the MRI assessment, with a focus on identification of early OC lesions in both the AECC and growth plate. Areas of chondronecrosis confined to epiphyseal cartilage, without involvement of overlying articular cartilage or subjacent bone, were classified as *OC‐latens*, while areas of chondronecrosis with corresponding delay of endochondral ossification were classified as *OC‐manifesta*. Healing lesions were defined as areas of retained necrotic cartilage surrounded by trabecular bone within the secondary ossification center (areas where endochondral ossification had resumed and bypassed areas of chondronecrosis). OC lesions of the growth plate were identified based on the presence of focal thickening of the growth plate extending into the metaphysis. Histologic lesions were manually coregistered to the 3D DESS images by alignment of landmarks including the growth plate profile and the articular surface curvature. The boundary of the bony epiphysis was defined on 0.5× magnified H&E‐stained photomicrographs and aligned to the matching boundary on 3D DESS MRI.

## 3 Results

**Figure 1:**
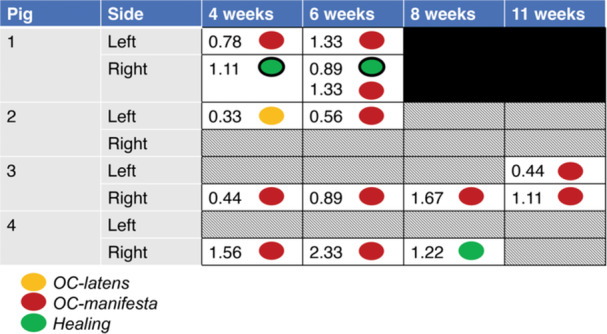
MRI examination findings by pig in the articular‐epiphyseal cartilage complex. Lesions noted at subsequent time points (ages) were in the same location and were presumed to represent the same lesion that was identified at the earlier time point. Lesions within the lateral humerus indicated with a black outline; all others lesions were medial. Numerical values indicate the size of the lesion in mm^2^.

**Figure 2:**
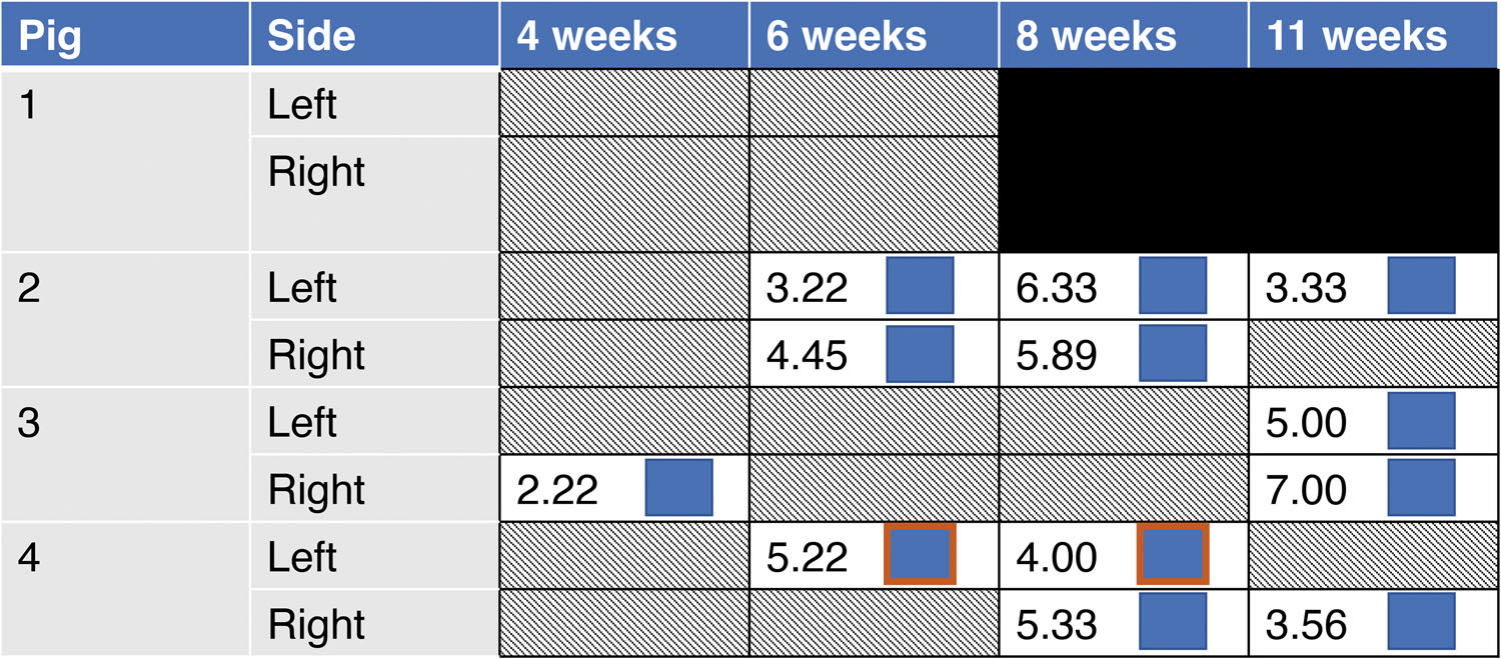
MRI examination findings by pig in the growth plate. Lesions noted at subsequent time points (ages) were in the same location and were presumed to represent the same lesion that was identified at the earlier time points. Lesions with an orange border were located laterally; all other lesions were medial. Numerical values indicate the size of the lesion in mm^2^.

Lesions identified by MRI are reported in Figure 1 (AECC) and Figure 2 (growth plate). All lesions were solitary at the reported location (AECC or growth plate) except for the right humerus from Pig #1 at 6 weeks of age, which had two lesions at different stages of development involving the AECC (one healing; one *OC‐manifesta*).

At 4 weeks of age, 5/8 humeri (4/4 pigs) had early OC lesions within the AECC as identified in MRI images. Pig #1 had a unilateral *OC‐manifesta* lesion in the left distal humerus and a healing lesion (cartilage surrounded by bone) within the secondary ossification center of the contralateral humerus. Pig #2 had a unilateral *OC‐latens* lesion. In its right humerus, Pig #3 had a small *OC‐manifesta* lesion and a small growth plate lesion, the only growth plate lesion observed at this time point. Pig #4 had a unilateral *OC‐manifesta* lesion.

**Figure 3:**
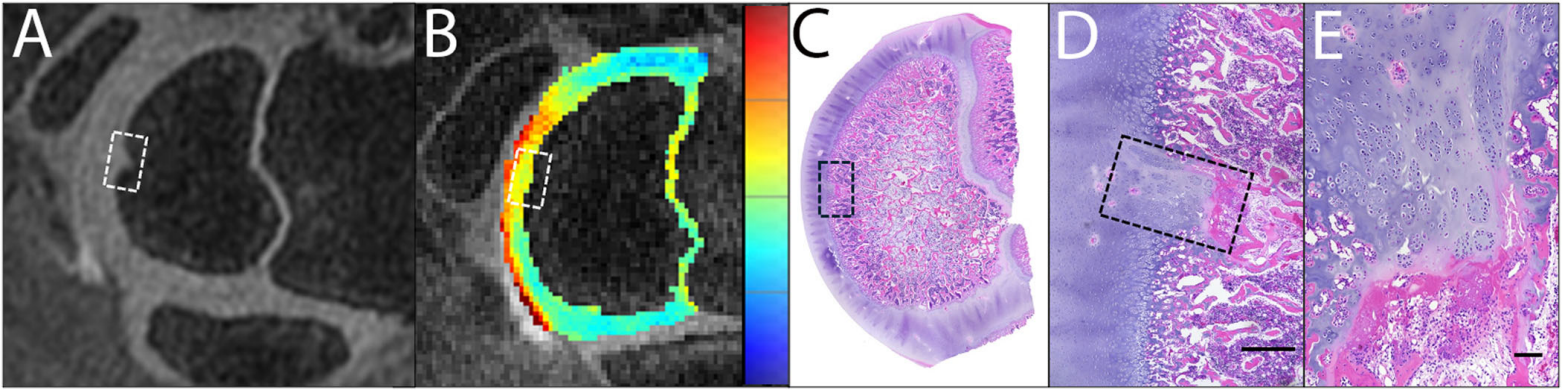
In Pig #1, at six weeks of age, an *OC‐manifesta* lesion was identified on both in vivo imaging with 3D DESS (A) and T2 mapping (relaxation time scale blue–red = 0–150 ms) (B) and ex vivo (C–E) in histological section, characterized by focal thickening of the AECC due to retention of partially necrotic cartilage. (C) H&E stained 0.5× magnification histological section with boxed area. (D) 4× magnification of *OC‐manifesta* lesion within the AECC, with necrotic debris and fibrin along the deep margin of the retained cartilage and mild subjacent myelofibrosis. Scale bar = 500 um. (E) 10× histological section showing chondrocyte clustering and small areas of necrotic chondrocytes within the retained cartilage. Scale bar = 100 µm.

**Figure 4:**
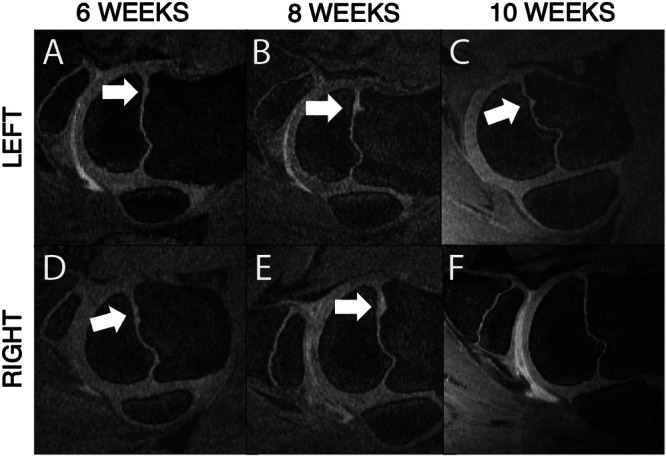
In Pig #2, 3D DESS allowed tracking of growth plate lesions over time, characterized by focal thickening of the growth plate cartilage at the same site over time. This pig had bilateral growth plate lesions (A, B, D, and E) that had resolved by 10 weeks on the right (F) and were in the process of resolution on the left (C) based on a reduction in size.

At 6 weeks of age, all AECC lesions identified at 4 weeks persisted at the same locations and showed signs of disease progression. Specifically, the three *OC‐manifesta* lesions observed at 4 weeks had all increased in size, and the *OC‐latens* lesion observed in Pig #2 progressed to an *OC‐manifesta* lesion. At this (6‐week) time point, the presence of an *OC‐manifesta* lesion in Pig #1 was also confirmed histologically (Figure 3). Newly observed growth plate lesions were identified bilaterally in Pig #2 (Figure 4) and unilaterally in Pig #4, whereas resolution of the growth plate lesion observed at 4 weeks of age was apparent in the right humerus of Pig #3.

**Figure 5:**
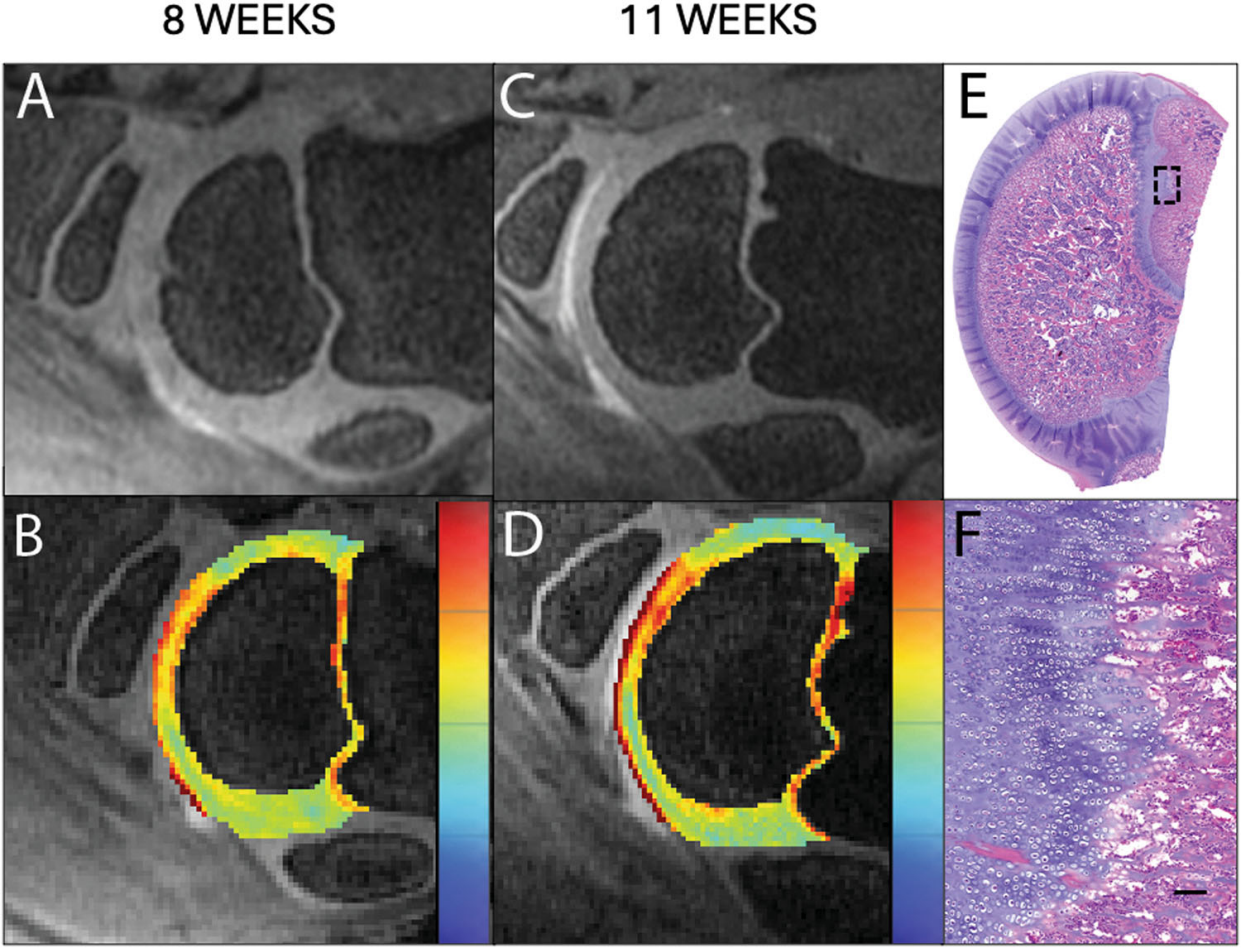
Pig #3 had an *OC‐manifesta* lesion in the AECC that was reduced in size from 8 (A and B) to 11 (C and D) weeks of age, as well as a growth plate lesion that was observed at 11 weeks on both 3D DESS and T2 mapping (relaxation time scale blue–red = 0–150 ms). (E and F) The growth plate lesion was confirmed histologically and consisted of focal thickening of the cartilage due to retention of proliferative and hypertrophic chondrocytes. 0.5x and 10x, H&E, scale bar (F) = 100 µm.

**Figure 6:**
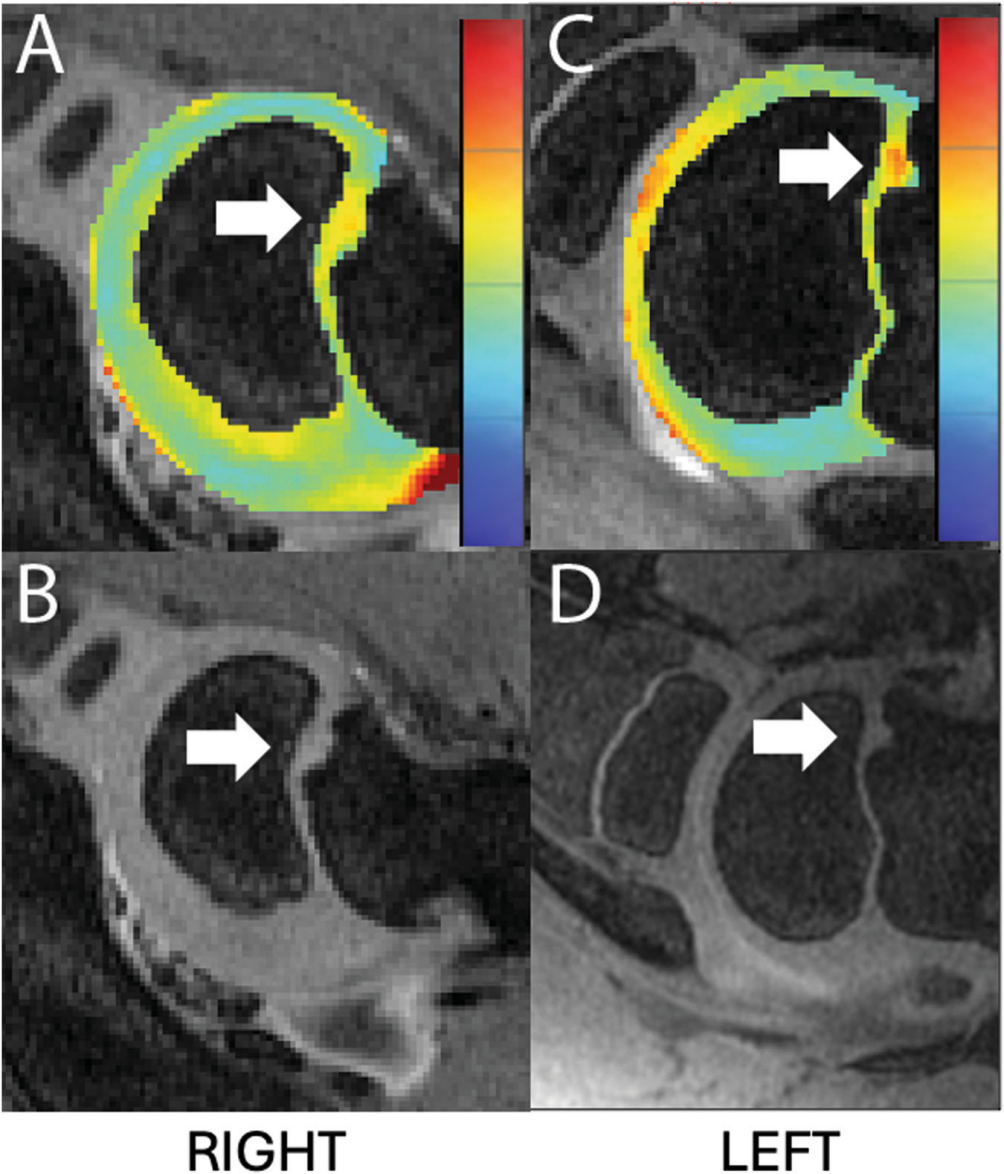
Pig 4 had a bilateral distal humeral growth plate OC at 8 weeks, with a medial lesion of the right humerus (A and B) and a lateral growth plate lesion of the left humerus (C and D), apparent on both 3D DESS and T2 (relaxation time scale blue–red = 0–150 ms).

At 8 weeks of age (3 pigs), Pig #2, with an *OC‐latens* at 4 weeks that progressed to an *OC‐manifesta* at 6 weeks, had complete resolution of this lesion. Conversely, both growth plate lesions persisted in this pig, but only one remained apparent at the time of the final, 11‐week scan. A unilateral *OC‐manifesta* lesion persisted across all imaging time points in Pig #3, which was subsequently confirmed histologically. Additionally, Pig #3 developed a new *OC‐manifesta* lesion by 11 weeks of age in the contralateral distal humeri that was inapparent at earlier scans (Figure 5). In this animal, growth plate lesions were also present bilaterally at the time of the final MRI and were confirmed histologically. In the right limb of Pig #4, a previously identified *OC‐manifesta* lesion was healing by 8 weeks and completely resolved by the final MRI evaluation at 11 weeks. Conversely, a growth plate lesion affecting the right leg remained apparent on the MRI examination at 11 weeks, unlike the lesion in the lateral portion of the left limb growth plate, which had resolved (Figure 6).

Lesions in the AECC (area subjacent to the ossification front only) ranged in size from 0.33 to 2.33 mm^2^ (i.e., 3–21 pixels), whereas lesions at the growth plate had a range in maximum area from 2.22 to 7.0 mm^2^ (i.e., 20–63 pixels). Most lesions were located at the medial aspect of the joint, except for a healing lesion of the capitellum observed in Pig #1 at 6 weeks of age and a unilateral growth plate lesion observed in Pig #4 at 6 and 8 weeks of age.

## 4 Discussion

Our findings demonstrate that quantitative and morphological MRI methods are effective in both the identification and longitudinal monitoring of naturally occurring OC lesions involving the distal humeral AECC and growth plate in juvenile pigs in vivo, detecting delays in endochondral ossification as small as 0.33 mm^2^ (i.e., 3 pixels). All pigs developed *OC‐latens* or *OC‐manifesta* lesions in the distal humeral AECC (unilateral 2/4, bilateral 2/4), with the earliest lesions observed at 4 weeks of age, which is earlier than previously reported [22, 27]. This may be in part due to the fact that most piglet studies begin assessment for OC at 7 weeks of age or later [12, 21]. One pig (Pig #3) had bilateral *OC‐manifesta* lesions at 11 weeks of age, consistent with previous reports of bilateral occurrence of both preclinical and clinical stage disease in children and animal species [3, 28]. Interestingly, one healing lesion (retained cartilage surrounded by bone) was identified at 4 weeks of age, suggesting that either these areas of retained cartilage are not associated with early OC lesions, or subclinical OC lesions may be present as early as at the time of birth [3].

Most prior animal model studies of OC/OCD have focused on the knee joint, investigating the histologic characteristics of lesions as well as describing unique features of the vascular supply that may predispose to lesion development [9, 20, 29, 30]. Investigation of OC/OCD at other predilection sites has been infrequently performed in animal models, although a recent study using susceptibility weighted MRI demonstrated that the vascular supply to the distal humeral OC/OCD predilection sites in young pigs bears a strong resemblance to that described for the distal femur [29, 31]. In the current study, we implemented MRI techniques previously used to explore induced lesions of OC/OCD in the knee joint and have found that they are also effective at identifying naturally occurring preclinical OC lesions in the AECC of the porcine elbow. These findings suggest that implementing these sequences in human patients may further our understanding of the pathophysiology, and specifically the factors contributing to the development and clinical progression of elbow OCD.

Our study is the first to identify OC lesions involving the growth plate using MRI. Similar to OC lesions in the AECC, growth plate lesions in the examined pigs were often present at earlier time points than previously reported and persisted at the terminal MRI scan. A previous study of physeal OC in the pig found 19%–100% (median 65%) of lesions at the growth plate identified by CT scanning had resolved by 180 days of age, when multiple growth plates were evaluated at multiple time points in a longitudinal study of 18 pigs [2]. These lesions were typically small (< 25% of growth plate affected), triangular in shape, and occurred in the metaphyseal‐side ossification front (as opposed to the epiphyseal margin), all of which held true for the lesions observed in this study.

While there is ample evidence that AECC OC has a similar pathogenesis in humans and animal species [32], growth plate (physeal) OC or a comparable condition is not described in the human literature. It is possible that this condition has not yet been recognized in humans, given the potential of spontaneous resolution and the need for cross sectional imaging to diagnose it at a young age. Alternatively, physeal OC may not occur in children due to differences in susceptibility associated with conformation or skeletal and/or vascular anatomy. Recent studies using CT to characterize physeal OC in pigs, along with our findings showing the utility of MRI to identify these lesions without ionizing radiation, provide strong impetus for future studies to explore their presence in humans [1, 2]. Indeed, children and animals both suffer from angular limb deformities and epiphysiolysis (separation of the epiphysis from the metaphysis due to a fissure through an abnormal growth plate), conditions where OC of the growth plate may be a contributing factor [10, 33].

While this study was limited in sample size, several lesions were present in the same location across time points, and a variety of outcomes were observed, including progression, persistence, and spontaneous resolution. Given that this study followed pigs longitudinally with in vivo imaging, early lesions were not confirmed histologically in most cases due to spontaneous resolution before euthanasia at 11 weeks of age, but the findings were consistent with those previously reported in pigs examined using similar techniques at ultrahigh field MRI [20]. The reported sizes of the observed lesions were limited by the resolution of the imaging scans and the AECC lesions were limited to measurement of the area of cartilage retained within the secondary ossification center only (excluding any abnormal overlying/preexisting AECC cartilage, which would not be distinguished on 3D DESS). Given that the spatial resolution was 0.33 × 0.33 mm^2^ with a 2 mm slice thickness, partial volume effects and potential signal averaging across slices may have underestimated the lesion size. Some of the smaller ROIs may span only 1–2 voxels in‐plane, which might introduce variability in quantification. Future studies with isotropic or thinner slices could mitigate this limitation. While the role of lesion size in progression to clinical disease has been suggested [30], the small size of the lesions identified in our study suggests that even those that were still present at the endpoints likely have gone on to heal versus progressing, but this remains an important area of future investigation. While previous studies have shown quantitative and morphological MRI to be effective in identifying OC lesions at ultrahigh field (10.5 T) at the distal humeral predilection site, effective application at a lower field strength (3 T) is essential for clinical translation [20]. We found that 3D DESS and T2 relaxation time mapping, used in tandem in this study, provide an effective approach to maximize the identification of lesions. Given the small size of early OC lesions, these techniques provide an opportunity for both quantitative assessment of the cartilage at early stages of disease, before structural changes to the AECC and resultant irreversible cartilage damage, and longitudinal tracking of disease progression in pediatric patients. Identification of multiple growth plate OC lesions was an unexpected, albeit important finding that demonstrates the suitability of MRI to serve as a noninvasive screening tool to identify the presence of similar lesions in children.

## Author Contributions

Study design: Ferenc Tóth. Data collection: Casey P. Johnson, Erick O. Buko, and Alexandra R. Armstrong. Data interpretation and processing: Ferenc Tóth, Casey P. Johnson, Erick O. Buko, and Alexandra R. Armstrong. All authors contributed to the drafting and revising of the manuscript.
